# Traumatisme fermé de l’abdomen occasionnant une pancréatite aiguë: présentation de cas

**DOI:** 10.11604/pamj.2018.30.126.14675

**Published:** 2018-06-13

**Authors:** Youssef Khaoula, Jawhar Mokni, Aloui Feten, Beizig Ameni, Maksoudi Chedly

**Affiliations:** 1 Service des Urgences Hôpital Régional de Kasserin, Tunisie

**Keywords:** Pancréatite aiguë, traumatisme abdominal, grave de pronostic, Acute pancreatitis, abdominal trauma, guarded prognosis

## Abstract

La pancréatite aiguë est une affection grave de pronostic réservé, son étiologie est soit biliaire ou alcoolique et l'origine traumatique est extrêmement rare et souvent associée à d'autres lésions intra abdominale. Nous présentons le cas d'un homme 56 ans aux atcds psychiatriques sous neuroleptiques et aux ATCDS de néphrotomie gauche et qui consulte dans les suites d'une chute d'un escalier 3 jours auparavant pour sd occlusif, à l'examen état hémodynamique respiratoire et neurologique correct, sensibilité épigastrique, une TDM pratiqué montre une pancréatite stade E traumatique avec au bilan de nombreuses perturbations: élévation des amylases, de la glycémie, des GB et de la CRP; l'évolution était défavorable avec le décès du patient 72 H plupart par une défaillance multi viscérale suite à un choc septique malgré une réanimation approprié. La pancréatite aiguë traumatique étant une affection grave, pourvue qu'elle est très rare et parfois difficile à diagnostiquer vue la non spécificité des signes cliniques, il faut savoir y penser afin la prendre en charge à temps.

## Abstract (en)

Acute pancreatitis is a serious disease having a guarded prognosis. Its etiology involves either bladder or alcohol. Traumatic origin is extremely rare and it is often associated with other intra-abdominal lesions. We here report the case of a 56-year old man with a history of psychiatric disorders for which he was under therapy with neuroleptics and of left nephrotomy. He presented, after a stair fall occurred 3 days before, with occlusive syndrome. Clinical examination showed normal respiratory hemodynamic and neurological status as well as epigastric tenderness. CT scan revealed traumatic pancreatitis stage E. Laboratory tests showed many disturbances: high amylase, blood glucose, whyte blood cell count and CRP. The patient died 72 hours after mainly because of multi-system organ failure secondary to septic shock, despite appropriate resuscitation. Acute traumatic pancreatitis is a serious condition. Although it rarely occurs and it is sometimes difficult to diagnose due to the non-specificity of clinical signs, physicians should suspect it in order to manage the disease early.

## Introduction

La pancréatite aiguë est une affection grave de pronostic réservé, son étiologie est soit biliaire ou alcoolique et l'origine traumatique est extrêmement rare et souvent associée à d'autres lésions intra abdominale.

## Patient et observation

**Figure 1:**
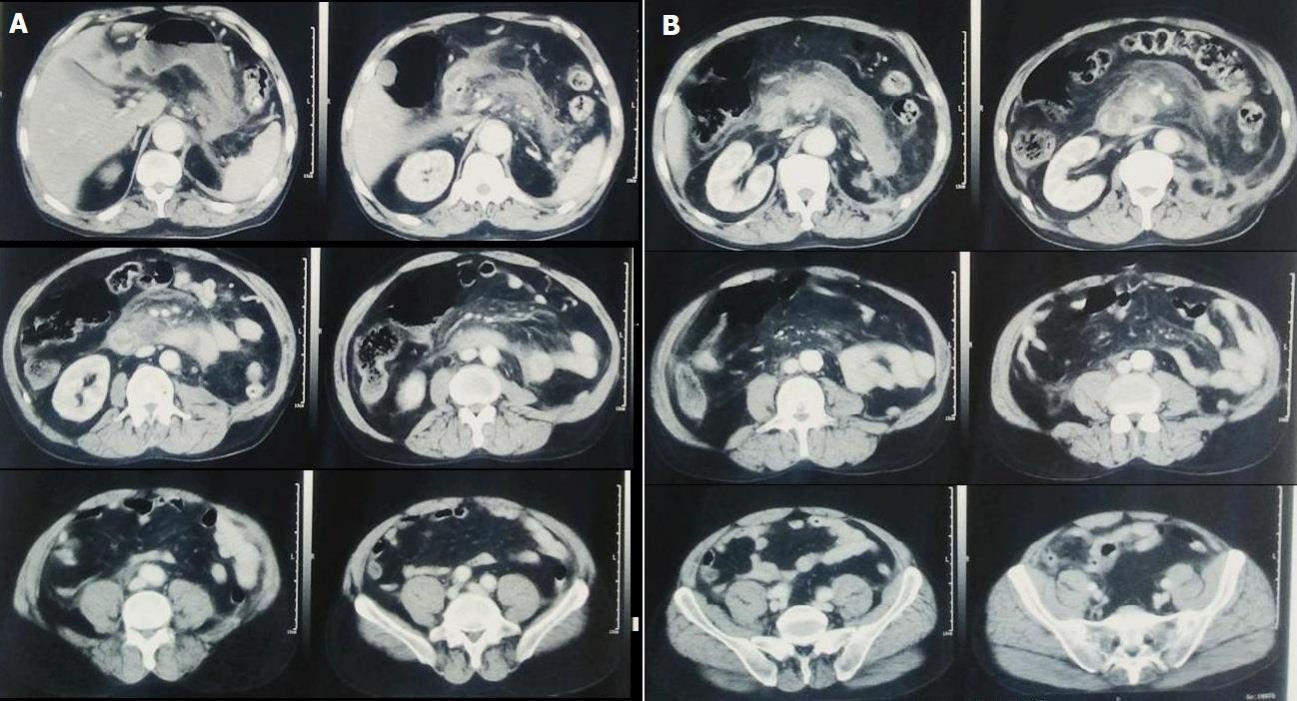
(A, B) image de TDM abdominale

C'est un homme âgé de 56 ans suivi en psychiatrie sous traitement neuroleptique et aux antécédents de néphrotomie gauche il y a une vingtaine d'année pour une étiologie indéterminée et qui consulte pour douleurs abdominales avec syndrome subocclusif. A noter la notion de traumatisme il y a 3 jours (chute d'escalier). Examen: état hémodynamique et respiratoire correct, état neurologique correct. Ecchymose périorbitaire gauche. Auscultation cardio-pulmonaire normale. Abdomen souple ballonné, sensibilité épigastrique. Le bilan biologique a montré de nombreuses anomalies: Glycémie élevé à 15,77 mmol, créat à 157, bilan hépatique normal, une CRP très élevé > 160 NFS: hb à 16,3 GB 22 400 plaq 164 000; TP à 62%. Une amylasémie 2 fois la normale à 345. TDM abdominopelvienne a montré (Figure 1 A, B): -un pancréas de densité hétérogène légèrement tuméfié siège d'une contusion hémorragique corpérocaudale avec épanchement péri pancréatique et importante densification de la graisse de l'arriere cavité des épiplons. -hémopéritoine de moyenne abondance diffus; péri hépatique, interanse, périsplénique et dans le douglas. -Absence d'anomalie d'allure traumatique du foie, de la rate et du rein droit. pancréatite stade E traumatique. La décision thérapeutique était basée sur l'approche du traitement conservateur et le patient a été hospitalisé en réanimation sous surveillance stricte clinique et biologique et principalement sous antibiothérapie large spectre forte dose et hyperhydratation. Malheureusement l'évolution était défavorable avec l'installation d'un choc septique suite à une surinfection des coulées de nécrose mal jugulée par une antibiothérapie à large spectre à forte dose et aboutissant à une défaillance multi viscérale et au décès du patient au bout de 72 heures de son admission.

## Discussion

La pancréatite aiguë est une affection grave pouvant mettre en jeu le pronostic vital. Son étiologie est souvent soit alcoolique soit biliaire. Vue sa localisation anatomique, l'origine traumatique est extrêmement rare en particulier en cas de traumatisme fermé, survenant seulement dans 0,2 à 4% des cas de traumatisme abdominal [1-4]. C'est une affection grave, de pronostic très réservé avec une mortalité très élevé, elle est souvent associé à d'autres lésions intra abdominale en particulier celle du duodénum; seule elle est extrêmement rare (0,1% des cas) [4]. Le diagnostic repose sur un faisceau d'argument clinique et biologique; l'imagerie permet de confirmer le diagnostic et de stadifier la pancréatite. Sur le plan clinique, elle se manifeste par des signes non spécifiques telque des douleurs épigastriques, des vomissements Sur le plan biologique, l'élévation de l'amylase n'est pas toujours présente et elle est non spécifique et non corrélée à la sévérité de l'atteinte; en effet l'amylase peut être normale le 1^er^ jour ou même dans les quelques jours qui suivent le traumatisme [2-5]. Sur le plan radiologique, la TDM abdominale reste le moyen le plus accecessible et le plus facile pour explorer un abdomen traumatique chez un patient hémodynamiquement stable; ce pendant, précocement faite le pancréas parait normal dans 20 à 40% des cas [2-5]. La mortalité est estimée à 9 à 46% des cas. Plusieurs facteurs s'y intriguent; le retard diagnostic ou de prise en charge semble en être un facteur déterminant, également la présence de lésions traumatiques associées, responsable notamment de choc hémorragique, ainsi que la pancréatite en elle-même et ses complications et elle n'est pas corolé au stade de la pancréatite [2-5]. Plusieurs facteurs s'associent pour prédire le pronostic en dehors de l'état hémodynamique du patient et l'atteinte du choledoque, les facteurs decrit dans le score de RANSON: la glycémie élevée, l'élévation de l'urée, l'hyperleucocytose sont tous des indicateurs d'un mauvais pronostic [6]. Pour finir, la prise en charge des lésions traumatiques du pancréas n'est pas codifié; chaque cas est particulier et plusieurs facteurs s'intriguent dans la décision thérapeutique: la nature de la lésion, sa localisation, l'atteinte ou non du cholédoque, les complications associés et l'état hémodynamique du patient sont les principaux arguments. Actuellement les nouvelles recommandations reposent sur l'approche du traitement conservateur non chirurgical [2, 3, 5].

## Conclusion

La pancréatite aiguë traumatique étant une affection grave, pourvue qu'elle est très rare et parfois difficile à diagnostiquer vue la non spécificité des signes cliniques, il faut savoir y penser afin la prendre en charge à temps.

## Conflits d’intérêts

Les auteurs ne déclarent aucun conflit d'intérêts.
